# Corrosion behaviour of welded low-carbon steel in the Arctic marine environment†

**DOI:** 10.1039/c8ra05371e

**Published:** 2018-08-28

**Authors:** Yoo Youl Choi, Myung Hyun Kim

**Affiliations:** R&D Center, Korean Register 36 Myeongji Ocean City 9-ro, Gangseo-gu Busan 46762 Republic of Korea yychoi@krs.co.kr +82-70-87998797; Department of Naval Architecture and Ocean Engineering, Pusan National University Busan 46241 Republic of Korea kimm@pusan.ac.kr +82-51-5102486

## Abstract

Arctic offshore sites have high potential for the exploration of energy resources; thus, data concerning the behaviour of structural materials in the Arctic environment are required. Here, we report the corrosive characteristics of welded low-carbon steels under simulated Arctic low-temperature conditions. The corrosion tendencies in the submerged and splash zones of offshore structures were investigated by immersion tests, salt spray tests (SST), and cyclic corrosion tests (CCT). The effects of decreasing seawater temperature on the corrosion were identified, and the differences in corrosion between the base metal (BM) and weld metal (WM) were analysed. In particular, the BM showed higher corrosion than the WM, indicating that the parent metal (PM) is corroded more than the fusion zone (FZ) in weld joints under severe corrosion conditions. Thus, we have identified the importance and influence of the thermal expansion of materials on corrosion under Arctic conditions.

## Abstract 2

The parent metal have shown higher corrosion than the weld zone under severe Arctic corrosion conditions.

## Graphical abstract

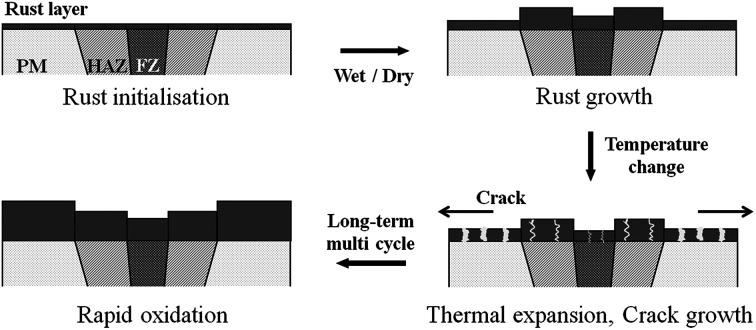

## Introduction

There is great uncertainty concerning future energy supply. However, the Arctic region has great potential for resource exploitation, and Arctic resources account for about 30% of global undiscovered natural gas and 13% of undiscovered global oil, mostly offshore and under less than 500 m of water.^1^ In the meantime, resource development has been delayed because of technical difficulties and the financial limitations of resource drilling. In recent years, the development of resources in the Arctic region has progressed in earnest because of the impact of global warming and the development of continuous drilling technology. Consequently, global oil companies and their partners are moving the offshore industry to Arctic regions. Therefore, appropriate material solutions for low-temperature Arctic applications are required to achieve the long-term durability of offshore structures.

Currently, most investigations into materials for Arctic use have focused on the mechanical properties of these materials in a low-temperature environment.^2,3^ These studies have mostly investigated the risk of brittle fracture in structural steels and, regarding the tensile properties, fracture toughness, arrest toughness, and fatigue.^2^ Unfortunately, the study of the corrosion characteristics under Arctic conditions for the prevention of structural corrosion is rare. This is because the corrosion of material in the Arctic region is generally expected to be less significant than that in temperate and tropical regions. In Arctic conditions, metallic corrosion is limited because the ice layer covering the metallic surface reduces oxygen access. Morcillo summarised the corrosion rates of metals exposed to the polar climate.^4^ The corrosion rate in the Antarctic zone was found to be only 0.10–0.87 μm y^−1^, and that in the Arctic zone has been measured to be 1.14–3.08 μm y^−1^ at different stations.^5–7^ However, the corrosion values were found to be higher, up to 222 μm y^−1^, when the measurement station was close to the sea.^7^ Therefore, we can predict that the corrosion of offshore structures is higher than previously reported. Because the monthly precipitation in winter and summer is low, 22 and 5 mm, respectively, in the Arctic region,^8^ salts on the metallic surfaces from seawater are rarely washed away by rain; thus, corrosion progresses continuously. Other studies have reported that electrochemical corrosion activity is possible below the ice layers.^9,10^ In addition, the splash zone suffers severe corrosion damage because of wave impact, the presence of sufficient oxygen, and the salt spray that continually wets and dries on the structure.^11–13^ Therefore, data concerning the corrosion of the splash zones of offshore structures is essential. In addition, the Arctic weather is becoming increasingly variable and unpredictable, showing high daily and monthly temperature variance.^14^ Therefore, information concerning the total corrosion impact in Arctic conditions is required.

Corrosion control in the Arctic region is a crucial technology, and safe and economical offshore structure design will be possible if accurate corrosion data can be acquired. Furthermore, protective coatings to prevent corrosion in the Arctic environment are currently under development, but this is challenging because organic materials change from elastic to plastic under low-temperature conditions and cracks in the coating can form because of iceberg impact.^15^ Therefore, consideration of possible corrosion is required for structural steels, and this preferable to the coating technique. Unfortunately, there are currently no studies of the corrosion of the steels used in the Arctic offshore structures in the submerged and splash zones.

In this study, we investigated the corrosion characteristics of welded low-carbon steel for offshore structures under Arctic low-temperature and marine conditions. First, the corrosion rates and corrosion trends were investigated in the submerged and splash zones under simulated conditions. Next, the difference in corrosion between the parent metal (PM) and the weld zone in the welded material was analysed.

## Experimental

Experiments were conducted using a base metal (BM) of YS460 MPa grade (FH460, Steel delivery condition H460TM), 100 mm thickness, Arctic offshore structural low-carbon steel. The steel was produced by the Thermo-Mechanical Controlled Process (TMCP), and the chemical composition range is shown in Table 1. Weld metals (WM) were prepared by flux cored arc welding (FCAW) and submerged arc welding (SAW), which are the most commonly used welding process for the production of ships and offshore structures. Welding materials of FCAW (Hyundai Welding Co. LTD., Trade name SC-81Ni2. Classification AWS A5.29 E81T1-Ni2C) and SAW (Hyundai Welding Co. LTD., Trade Name: H-14, Classification AWS A5.17 EH14) with sufficient toughness under the low-temperature environment were selected, and the chemical composition of the welding material is described in Table 1. WM specimens were sampled from at the quarter thickness (*t*/4) location, and each specimen contained fusion zone (FZ, weld metal), heat affect zone (HAZ), and PM (unaffected base metal) regions. The sizes of the BM and FCAW specimens were 100 mm (*W*) × 30 mm (*L*) × 5 mm (*T*), whereas the SAW specimen measured 120 mm (*W*) × 30 mm (*L*) × 5 mm (*T*) because of the wider FZ area. Detailed information of specimen preparation on K-groove weld joint design and cutting direction are provided in the ESI (Fig. S1†). The total surface of each specimen was grinded with #2000 grade of SiC sandpaper. After washing the surface with deionised water and acetone, ultrasonic cleaning was performed, and the specimens were dried and weighed in the range of 0.1 mg.

**Table 1:** Chemical composition of the experimental base metal (BM) and welding materials (wt%)

	C	Si	Mn	P	S	Others	Fe
Base metal	0.06–0.10	0.05–0.30	1.20–1.60	≤0.012	≤0.003	Cu, Ni, Nb, Ti, Ca	Bal.
FCAW wire	0.05	0.26	1.27	0.01	0.01	Ni (2.23)	Bal.
SAW weld metal	0.06	0.13	1.37	0.016	0.007	—	Bal.

To investigate the corrosion properties, corrosion tests on BM and WM were conducted by immersion, salt spray (SST), and cyclic corrosion (CCT) tests. The corrosion conditions in Table 2 simulate the submerged zone and the splash zone environment. The immersion tests and the SST were based on international standards. In the case of CCT, the Hyundai/Kia MS 600-66 (CCT C) industry standard, based on the study of Matsuoka *et al.*^16^ and a simulated low-temperature environment, was utilised because there is no international standard for CCT with controlled climate and freezing condition. Seawater was simulated using a 3.5% NaCl solution. After the corrosion tests, the corrosion products on the surface were washed with deionised water and removed by chemical cleaning procedures described in the ASTM G1 standard.^17^ In the case of the thick layer of corrosion products, partial blasting was performed before the chemical cleaning process. After removing the corrosion products from the surface, the specimens were dried and weighed.

**Table 2:** Detailed experimental conditions and applied test standards for each corrosion test

Test item	Test specimen	Test condition	Test standard
Immersion test	Bare metal, FCAW, SAW	3.5% NaCl solution, solution temperature: 2 °C, 15 °C, experiment time: 240 hours	ASTM G 31
Salt spray test	Bare metal, FCAW, SAW	5% NaCl solution, test temperature: 35 °C, experiment time: 240 hours	ISO 9227
Cyclic corrosion test	Bare metal, FCAW, SAW	5% NaCl solution, experiment time: 240 hours (20 cycles), test condition (1 cycle): salt spray (35 °C, 95% R.H.) 4 h → dry (70 °C, 30% R.H.) 2 h → wet (50 °C, 95% R.H.) 2 h → dry (25 °C, 60% R.H.) 1.5 h → low temperature (−40 °C) 2.5 h	Hyundai/Kia MS 600-66 (CCT C)

Immersion test was conducted in a constant-temperature seawater solution using a constant-temperature water bath (Julabo TW20). SST and CCT experiments were conducted following AT2600IP (ASCOTT), and more than five test specimens were used for each experiment, and the total average value was used as a result. The SST and CCT equipment was verified in advance through official calibration to prevent corrosion deviations arising from the location on the specimen. The calculations of the corrosion rates for immersion tests, SST, and CCT are shown in the ESI.† To investigate the microstructure, specimens were etched by a 2% nital solution, and the surfaces were observed using a metallurgical microscope. The corroded surface chemical composition changes were measured *via* scanning electron microscopy energy-dispersive spectroscopy (SEM-EDS, JEOL, JSM-6701F, acceleration voltage 15.0 kV, Oxford EDAX (INCA-Penta FET X3)). To measure the coefficient of thermal expansion (CTE), all specimens were cut to a size of 5 mm × 5 mm × 5 mm, and measurement followed the ASTM E831 method^18^ utilising thermomechanical analysis (TMA, TA Instruments, TMA-Q400). In the case of the FCAW and SAW specimens, the TMA samples were extracted from the FZ.

## Results

**Fig. 1:**
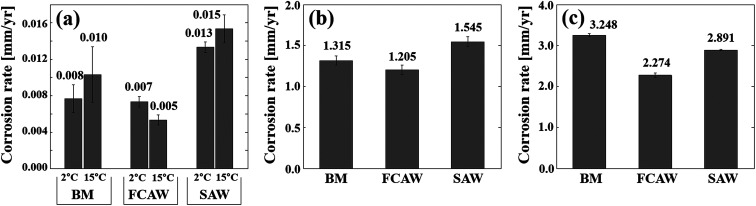
Corrosion rates of experimental specimens obtained from various corrosion tests. (a) Immersion test, (b) salt-spray test, and (c) cyclic corrosion test.

Corrosion tests were performed on BM and WM specimens to evaluate the corrosion characteristics of the low-carbon steel in an Arctic offshore environment. Fig. 1 shows the test results for the immersion test, the SST, and the CCT. The immersion results are based on seawater immersion conditions, and SST and CCT results are based on salt spray conditions. The Det Norske Veritas group (DNVGL) defined as the climatic region based on the seawater temperature (*T*) of the surface water, for example, Arctic region, *T* ≤ 7 °C; temperate region, 7 °C < *T* ≤ 12 °C; sub-tropical region, 12 °C < *T* ≤ 20 °C; and tropical region. *T* > 20 °C.^19^ Therefore, in the immersion tests, 2 and 15 °C were set as the representative temperatures, and the corrosion differences according to the climate were compared. In this figure, only the final corrosion rates are shown, and detailed test data is given in the ESI.†

Concerning the immersion test results (Fig. 1a), the corrosion rate of all specimens showed a satisfactory value of 0.005–0.015 mm y^−1^. The degree of corrosion was in the order of FCAW < BM < SAW, and the difference between BM and FCAW was very small. The BM specimen showed a slight higher corrosion rate than FCAW but lower than SAW, thus the degree of corrosion was in the order of FCAW < BM < SAW. As the seawater temperature decreased, no noticeable decrease in corrosion was observed. No pitting corrosion was found, and partial red-brown general corrosion was observed.

In the salt spray tests (Fig. 1b), the corrosion rate was 1.205–1.545 mm y^−1^, which is 70 times higher than that measured in the seawater immersion tests. This indicates that the base material is very weak in salt spray exposure conditions. The corrosion tendency was similar to the results of immersion test in the order of FCAW < BM < SAW. In general, SST is less relevant to reproduce practical corrosive conditions, but tests were conducted to compare corrosion differences between specimens under constant temperature conditions in saline conditions and for comparison with the CCT test results.

The CCT test was conducted by lowering the exposure temperature to −40 °C after the salt spray step. As shown in Fig. 1c, serious corrosion rate in the range of 2.274 to 3.248 mm y^−1^ was confirmed. The degree of corrosion was in the order of FCAW < SAW < BM, and the formation of a significant rust layer was visually observed (ESI†). The corrosion level determined from the CCT was more than 130 times higher than the seawater immersion test, and it was confirmed that the corrosion change of the steel was caused by the repeated wetting and drying, as well as temperature changes. In particular, by comparing the results shown in Fig. 1a and b we confirmed the specific corrosion of the BM is slightly higher than FCAW but much lower than SAW. This is important information for base metals and weld joints in the design of the offshore materials; thus, we identified the causes.

## Discussion

As shown in Fig. 1, the corrosion trends for the BM and the WM under Arctic conditions were confirmed. In the submerged simulated condition test, rust was produced in the form of general corrosion, but it was difficult to visually distinguish the corrosive difference between the BM and the WM specimens because of the small amount of corrosion. However, when comparing the corrosion rates *via* measuring the weight loss, the values for the WM specimens were generally higher than those of the BM specimens. This indicates that the corrosion influence on FZ or HAZ is relatively higher than those of the PM. In addition, no significant decrease in corrosion was observed, even when the seawater temperature was reduced from 15 to 2 °C. In previous studies, it has been shown that corrosion increases as seawater temperature increases.^20^ However, in our experiment, the temperature difference is small, and the increased amount of dissolved oxygen at low temperature has maintained the corrosiveness. In addition, as seawater temperature changes, an inverse correlation exists between the diffusion rate of oxygen molecules and the amount of dissolved oxygen; thus, we cannot predict the reduction in corrosion as the seawater temperature decreases.

On comparing the overall corrosion tendency, we found that the BM was less corroded in most experiments, but, from the CCT results, the BM specimens showed the most corrosion. This means that more corrosion can occur in the PM region than the FZ region in the splash zone. Therefore, it is necessary to find the cause of this phenomenon for the design of corrosion-resistant Arctic offshore structures. Thus, the influence of corrosion factors on the corrosion was investigated by analysing the microstructure, chemical composition, and CTEs.

**Fig. 2:**
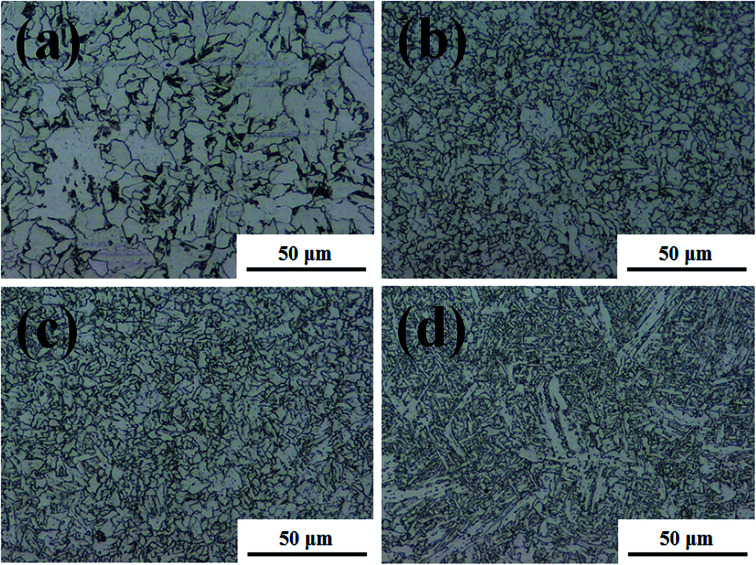
Microstructural characterisation of the welded joint of FCAW specimen. (a) Parent metal (PM), (b) coarse grain heat affected zone (CGHAZ), (c) fine grain heat affected zone (FGHAZ), and (d) fusion zone (FZ).

**Fig. 3:**
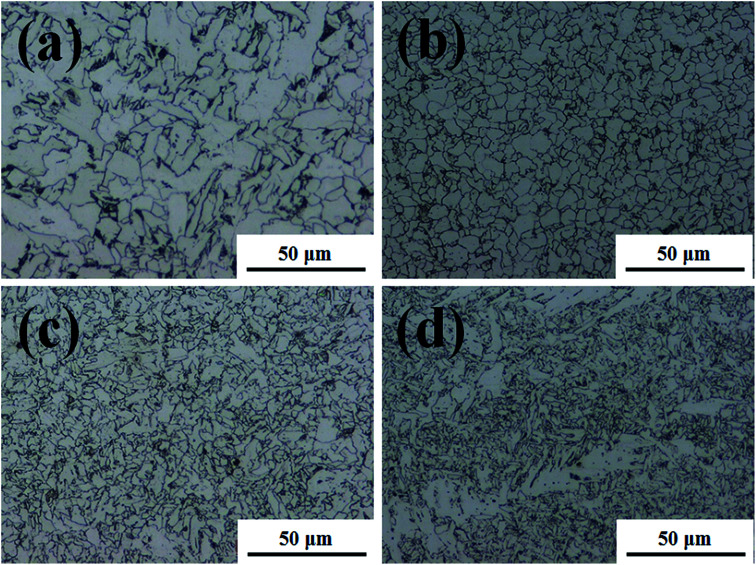
Microstructural characterisation of the welded joint of SAW specimen. (a) Parent metal (PM), (b) coarse grain heat affected zone (CGHAZ), (c) fine grain heat affected zone (FGHAZ), and (d) fusion zone (FZ).

The microstructures were analysed to explain the corrosion of the BM. This is because local differences in microstructure, such as grain size and elemental microcomposition, can give rise to different corrosion properties. Therefore, the microstructure can be used to compare the corrosion difference between the FZ and PM in the specimen. Fig. 2 and 3 exhibit the microstructure morphology of FZ, coarse grain heat affected zone (CGHAZ), fine grain heat affected zone (FGHAZ), and PM in the FCAW and SAW test specimens. In the case of the FCAW test specimen, the PM phase was mainly quasi-polygonal ferrite + acicular ferrite + pearlite, FGHAZ was bainitic ferrite + granular bainite + acicular ferrite, CGHAZ was granular bainite + martensite + acicular ferrite, and FZ was acicular ferrite + martensite. Ralston and Birbilis reported that a finer microstructure gives rise to a more reactive surface as a result of the smaller grains and more grain boundaries, making the surface more prone to corrosion.^21–23^ In addition, bainite is nobler than ferrite; thus, a ferrite–bainite microstructure results in galvanic corrosion.^24^ In practice, the coexistence of ferrite and bainite phases in the HAZ is expected to result in more corrosion than in the FZ (ferrite + martensite) and PM (ferrite + pearlite). Considering the grain size, HAZ was found to have smaller grains than the PM, and the WM was found to have a dense needle-like structure. Bhagavathi reported that a ferritic–martensitic microstructure shows decreased galvanic effects because of the similarity between BCC and BCT crystal structures.^25^ However, it has been reported that the corrosion rate of ferrite–martensite is higher than that of the ferrite–pearlite phase;^26^ thus, the corrosion of the FZ would be higher than that of the PM and lower than that of the HAZ. Therefore, considering the microstructure, it is expected that corrosion progresses in the order of PM < FZ < HAZ in the welding joint of FCAW. In the case of the SAW test specimen, the PM phase was mainly quasi-polygonal ferrite + acicular ferrite + pearlite, FGHAZ was quasi-polygonal ferrite + acicular ferrite + granular bainite, CGHAZ was quasi-polygonal ferrite + acicular ferrite + granular bainite, and FZ was granular bainite + quasi-polygonal ferrite + acicular ferrite. Based on observation, HAZ has the smallest grain size and shows a ferrite–bainite structure with a high ferrite ratio. Therefore, it is expected that the corrosion rate of HAZ and FZ will be higher than that of PM because of the galvanic couple corrosion of ferrite–bainite microstructure. Consequently, if we predict the corrosion tendency of the structures, the corrosion of the HAZ should be the worst, and the corrosion resistance of the PM should be highest. In fact, several reports have shown that HAZ suffers severe localised corrosion attack relative to the PM and FZ, which is attributed to the microstructural characteristics of these regions.^27,28^ Thus, we tried to investigate the corrosion difference of regions in the weld joint by comparing the chemical compositions of each metallic surface.

**Fig. 4:**
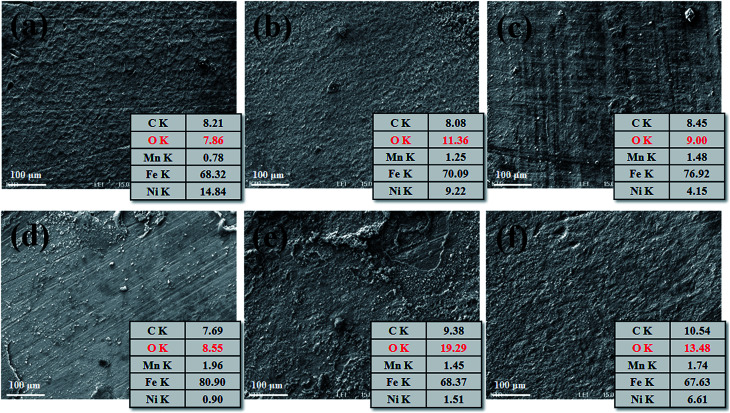
SEM and EDS characterisation of the corroded surface of (a) FCAW-PM, (b) FCAW-HAZ, (c) FCAW-FZ, (d) SAW-PM, (e) SAW-HAZ and (f) SAW-FZ specimens after one cycle of CCT (wt%). The EDS data were obtained over the entire area shown in each SEM image.

Fig. 4 shows the surface morphology and EDS analysis results of FCAW and SAW specimens. In general, rust is comprised of various types of oxides: hydrated oxides, oxyhydroxides, and miscellaneous crystalline and amorphous substances, but all components are mainly composed of iron and oxide.^29^ Therefore, measuring the oxygen ratio in the rust layer can provide information about the progression of corrosion under equal exposure conditions. Therefore, to understand the initial corrosion, the chemical composition of the rust layer was analysed at representative positions but excluding the severely corroded region after one cycle of CCT. As shown in Fig. 4, general corrosion occurred over the entire area. In both case of FCAW and SAW, corrosion progressed in the order of PM < FZ < HAZ. Therefore, we confirmed that the corrosion of the PM region progresses the least during the initial corrosion stage.

Next, initial and long-term corrosion tests were carried out to determine whether the corrosion tendency can change. The corrosion rates of BM, FCAW, and SAW were approx. 4.05, 5.26, and 6.98 mm y^−1^, respectively, at the start of a CCT cycle. In contrast, the corrosion rates of BM, FCAW, and SAW were approx. 3.25, 2.27, and 2.89 mm y^−1^, respectively, after one full cycle of CCT. The overall decrease in the corrosion rate suggests that the corrosion delay was due to the oxide layer. Here, it can be seen that the BM specimen with the smallest amount of corrosion at the beginning becomes the most corroded in the long-term stage. In practice, the corrosion layer in the PM region was the thickest of all the regions after 20 cycles of CCT. Therefore, it can be seen that the corrosion of the PM region is relatively significant after a certain corrosion period.

**Fig. 5:**
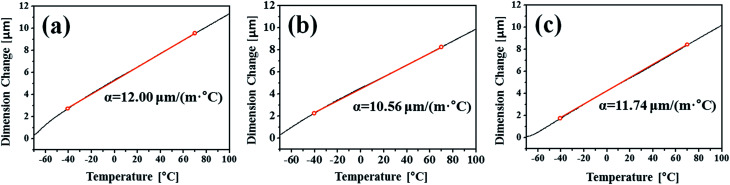
TMA curves and the coefficient of thermal expansion (CTE) of samples of the (a) base metal (BM) and (b) fusion zone (FZ) of the FCAW specimen and the (c) FZ of the SAW specimen.

**Fig. 6:**
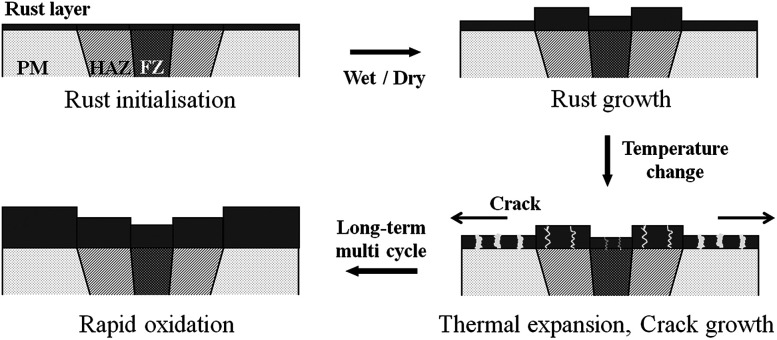
Schematic of the rapid oxidation of the parent metal (PM), heat affected zone (HAZ), and fusion zone (FZ) under CCT conditions.

The CCT was characterised by adding a temperature change condition to the SST in consideration of the Arctic environment. In fact, comparing the corrosion in the splash and submerged zones, the splash zone condition is more severe considering the repeatability of the wetting/drying conditions arising from wave action and the daily temperature difference depending on the wind speed and seasonal differences. Therefore, thermal expansion measurements were conducted to confirm whether the temperature change affects the corrosion tendencies of the specimens. Fig. 5 shows the CTE measurement data from −50 to 100 °C, where the CTE is calculated based on the practical experiment range. As a result, the values for the BM, FCAW, and SAW were 12.00, 10.56, and 11.74 μm (m °C)^−1^, respectively. Thus, the CTE of BM was highest. Because the CTEs of iron oxides are less than 8.5 μm (m °C)^−1^ below 100 °C,^30^ thermal stress occurs between the oxide and metallic layers. Therefore, it is expected that cracks will occur in the iron oxide layer at the locations where the stress is concentrated during corrosion. When the oxide layer on the surface is thick, the layer is damaged by the relatively high thermal deformation of the BM, and continuous corrosion will progress around the damaged locations. Fig. 6 illustrates the schematic corrosion progression pattern in the WM through the CCT. After the initial rust layer is formed, corrosion progresses in the order of PM < FZ < HAZ, depending on the corrosion potential. However, after a certain thickness of rust layer has developed, cracks with relatively high density occur in the PM, which ensures the most stress because of repeated temperature changes. These cracks provide paths for water, oxygen, and other corrosive substances to enter and cause additional corrosion. The protective density and adherence of the rust layer depend on the duration wetness;^29^ thus, the existence of marine components (chloride) and long relatively long wet/dry cycles may produce unstable rust layers that possess many defects (holes and cracks). The defects inhibit the evaporation of trapped water; therefore, rapid oxidation occurs and reduces the compactness of the rust layer on carbon steel.^31^ Thus, when corrosion progresses in the medium-and long-term, corrosion mostly occurs in the PM as a result of the influence of stress due to the differences in the CTE of each specimen. This means that the importance of impact on the corrosion rate is primarily affected by the compactness and adhesiveness of the rust layer than any other factors under severe corrosion conditions. Although many studies have dealt with the rust-expansion-induced cracking of steel bars in reinforced concrete structures,^32,33^ no studies have shown the different in rust cracking between the PM and FZ in weld joint regions.

Consequently, it can be seen that a difference in CTE is a critical factor for the corrosion behaviour under heavy corrosion conditions. It also shows the possibility of significant corrosion progression by repeated rust layer cracking. Therefore, this study suggests the severe influence of corrosion on PM in weld joints, and further related research will be needed in the future.

## Conclusions

We investigated the corrosion characteristics of welded low-carbon steel for Arctic offshore structures under low-temperature conditions. Immersion tests, SST, and CCT were conducted in seawater under low-temperature and low air temperature conditions. The following conclusions were obtained.

(1) Concerning the immersion test that simulates the submerged conditions, the corrosion rate of all specimens showed a value of 0.005–0.015 mm y^−1^. The BM specimen showed a slight higher corrosion rate than FCAW, but lower than SAW.

(2) Through the SST and CCT tests that simulate the splash zone conditions, all specimens showed much higher corrosion rate of 1.205–1.545 mm y^−1^ and 2.274–3.248 mm y^−1^ respectively than the immersion test, which indicated that the base material is weakened in splash zone conditions. In particular, the CCT tests resulted in the most corrosion in the BM specimen, and these phenomena led to more corrosion at the PM than the FZ in the WM specimens.

(3) Through the medium-and long-term CCT experiments, it was confirmed that the corrosion tendency of the PM region reversed as corrosion progressed. This phenomenon was confirmed by measuring the change in the chemical composition of the oxide layers in the WM specimens, where the corrosion rate was lowest in the first cycle of CCT but was highest after a full CCT cycle. Accordingly, rapid oxidation in the PM is expected because of the presence of cracks and voids in the oxide layer. From the analysis of the measured CTE, it is predicted that the stress in the oxide layer in the BM region will be stronger than in other regions because of the high CTE. Therefore, under conditions of repeated temperature change, cracks in the rust layer induced by thermal expansion occur and rapid corrosion progresses because of the easier diffusion of oxygen and moisture.

This study shows the reversed corrosion on welded materials in heavy Arctic marine corrosion conditions because of the occurrence of cracks in the rust layer. Therefore, in the case of splash zones, the influence of corrosion in the PM can be more important than in the FZ. Because the specification of corrosion allowance and welding design must consider the criticality of the structure, we expect that the results of this investigation will be important for the safe and long-term design of Arctic offshore structures.

## Conflicts of interest

There are no conflicts to declare.

## Supplementary Material

RA-008-C8RA05371E-s001
