# Supplementary material for: Corrosion behaviour of welded low-carbon steel in the Arctic marine environment
Source: RSC Adv. 2018 Aug 28;8(53):30155–62. doi: 10.1039/c8ra05371e (PMC9085398; doi:10.1039/c8ra05371e)
Supplement: RA-008-C8RA05371E-s001 [file RA-008-C8RA05371E-s001.pdf]

Electronic Supplementary Material (ESI) for RSC Advances.
This journal is © The Royal Society of Chemistry 2018

RSC Advances

Supplementary Information

Corrosion behaviour of welded low-carbon steel in the Arctic marine environment

Yoo Youl Choi* and Myung Hyun Kim

* *Author for correspondence:*
Dr. Yoo Youl Choi
Phone: +82 70 8799 8797
E-mail: yychoi@krs.co.kr

Supplementary Information Figures and Tables

Specimen preparation

In this study, weld joints with a K-groove were selected because of their excellent mechanical properties and welding workability based on fully pre-welded performance tests of structural steel for Arctic offshore structures. The thick 100-mm rolled steel for welded structures was welded by a professional welder, and non-destructive testing (NDT) was performed to determine the presence of internal defects. After confirming the quality of the weld zone, all specimens were cut at the quarter thickness location and each specimen dimension was checked. The specimens were then subjected to surface grinding and cleaning for corrosion tests.

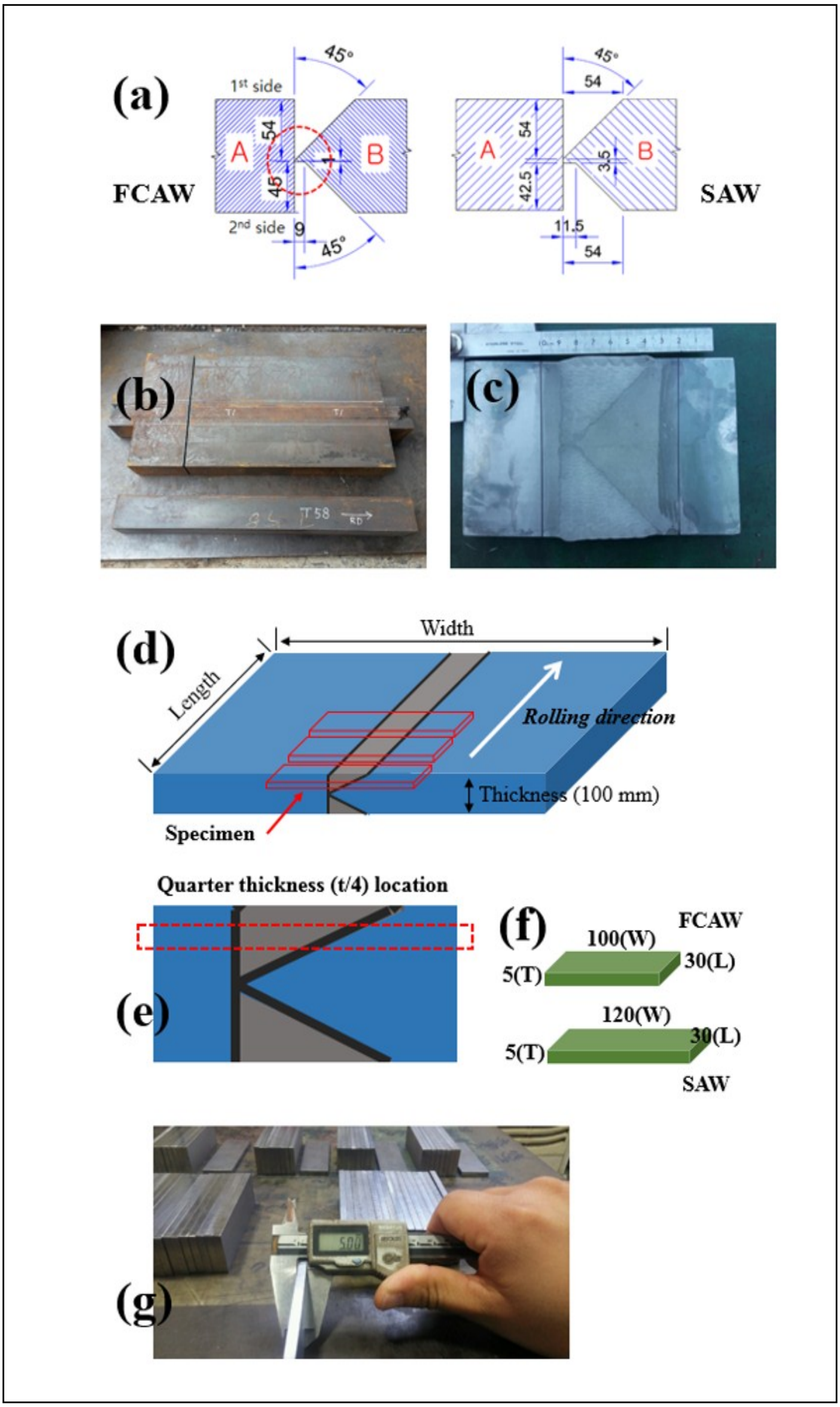

Figure S1. Figures showing the preparation process of the specimen.
(a) K-groove weld joint design for FCAW and SAW.
(b) Photograph of a plate of welded metal prepared by the FCAW process.
(c) Cross-sectional photograph of the weld joints prepared by the FCAW process.
(d) Diagram showing the cutting direction of specimens from the welded plate.

This journal is © The Royal Society of Chemistry 20xx

Please do not adjust margins

(e) Diagram showing the cutting location of specimens from the welded plate.
(f) FCAW and SAW specimen dimensions.
(g) Checking of specimen dimensions after cutting.

To ensure reliability, the immersion test, the SST, and the CCT used more than five test specimens, and the total average value was used as a result. In the result of CCT, the measurement result has shown small deviations arising from the location on the specimen. The corrosion rate of the immersion test, the SST and the CCT were calculated using the following equation:

$R_c = 87.6\ \Delta W / \rho A T,$

where ρ is the metal density [g cm^{-3}], ΔW is metal weight loss rate [mg cm^{-2}], A is the exposed area [cm^2], T is the exposure time [h], and R_c is the average corrosion rate [millimetres per year].

Table S1. Corrosion rates of experimental specimens obtained from the Immersion test.

Test condition	Specimen	Avg. weight loss [g]	Avg. surface area [cm^2]	Weight loss per unit area [g/m^2]	Corrosion rate [mm/yr]
2°C 3.5% NaCl Solution	BM	0.012	72.61	1.6525	0.008
	FCAW	0.011	72.55	1.5115	0.007
	SAW	0.024	86.16	2.8049	0.013
15°C 3.5% NaCl Solution	BM	0.016	72.61	2.1800	0.010
	FCAW	0.009	72.55	1.1669	0.005
	SAW	0.028	86.16	3.2964	0.015

Table S2. Corrosion rates of experimental specimens obtained from the salt spray test.

Specimen		Surface area [cm^2]	Weight loss per unit area [g/m^2]	Corrosion rate [mm/yr]
BM	No.1	72.59	289.43	1.342
	No.2	72.13	266.57	1.236
	No.3	72.39	284.58	1.319
	No.4	72.42	298.34	1.383

Please do not adjust margins

Please do not adjust margins

	No.5	72.34	279.46	1.295
	Average	73.37	283.67	1.315
FCAW	No.1	72.18	247.03	1.145
	No.2	72.10	248.10	1.150
	No.3	72.28	267.43	1.240
	No.4	72.29	275.04	1.275
	No.5	72.05	261.65	1.213
	Average	72.18	259.85	1.205
SAW	No.1	86.11	318.11	1.475
	No.2	86.59	325.40	1.508
	No.3	86.62	337.84	1.566
	No.4	86.54	352.99	1.636
	No.5	86.44	332.44	1.541
	Average	86.46	333.36	1.545

Table S3. Corrosion rates of experimental specimens obtained from the cyclic corrosion test.

Specimen		Surface area [cm^2]	Weight loss per unit area [g/m^2]	Corrosion rate [mm/yr]
BM	No.1	72.64	708.70	3.285
	No.2	72.09	695.61	3.225
	No.3	72.46	703.31	3.260
	No.4	72.43	708.69	3.285
	No.5	72.38	713.05	3.305
	No.6	72.88	701.74	3.253
	No.7	72.07	687.65	3.188
	No.8	72.19	692.49	3.210

This journal is © The Royal Society of Chemistry 20xx

Please do not adjust margins

Please do not adjust margins

	No.9	72.33	694.66	3.220
	Average	72.39	700.66	3.248
FCAW	No.1	72.34	468.76	2.173
	No.2	72.16	498.36	2.310
	No.3	72.17	495.83	2.298
	No.4	72.23	488.79	2.266
	No.5	72.09	481.93	2.234
	No.6	72.34	484.61	2.246
	No.7	72.4811	496.67	2.302
	No.8	72.13	503.67	2.335
	No.9	72.20	496.96	2.304
	Average	72.24	490.62	2.274
SAW	No.1	86.21	617.25	2.861
	No.2	86.18	628.25	2.912
	No.3	86.30	626.40	2.904
	No.4	86.28	616.85	2.859
	No.5	86.25	625.82	2.901
	No.6	86.27	623.19	2.889
	No.7	86.33	627.12	2.907
	No.8	86.36	623.48	2.890
	No.9	86.30	624.56	2.895
	Average	86.27	623.66	2.891

This journal is © The Royal Society of Chemistry 20xx

Please do not adjust margins

Please do not adjust margins

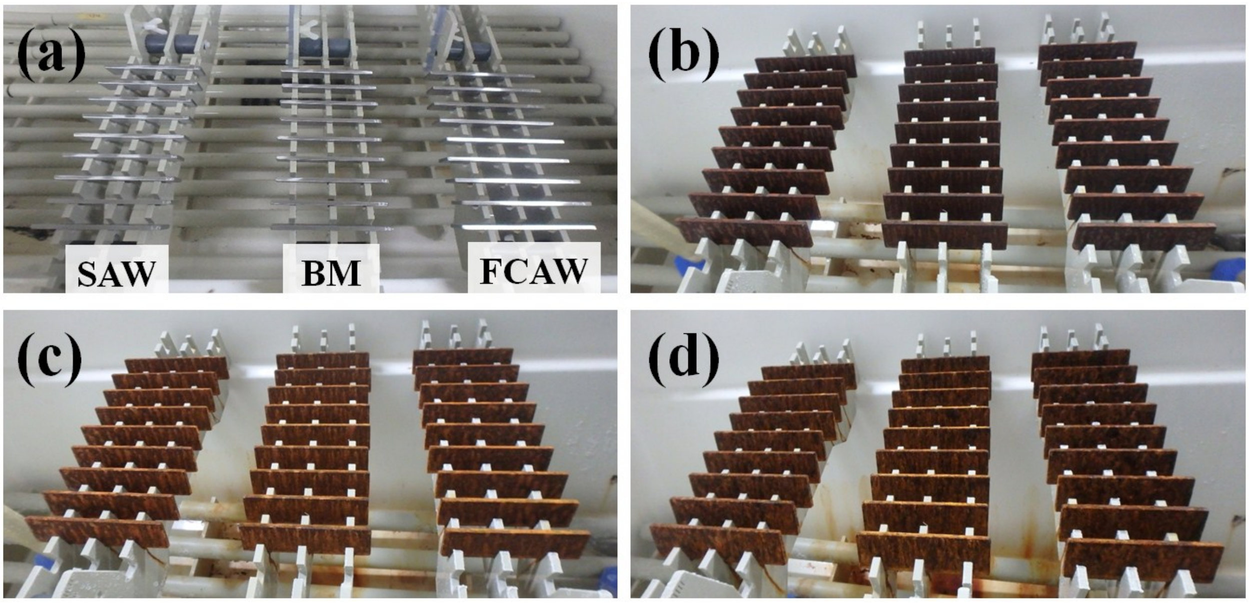

Figure S2. Photograph of BM, FCAW, SAW specimens of (a) before, (b) 6 cycles, (c) 12 cycles, and (d) 18 cycles of CCT.

This journal is © The Royal Society of Chemistry 20xx

Please do not adjust margins
